# Phase Transformation‐Assisted Nucleation and Growth of a Single‐Phase FeCoNiCuNb Alloy

**DOI:** 10.1002/advs.202509237

**Published:** 2025-11-06

**Authors:** Zhimin Guo, Fuchen Zhou, Jinhua Yu, Jun Ding, Evan Ma, Qian Yu

**Affiliations:** ^1^ Center of Electron Microscopy and State Key Laboratory of Silicon Materials Department of Materials Science and Engineering Zhejiang University Hangzhou 310027 China; ^2^ Center for Alloy Innovation and Design State Key Laboratory for Mechanical Xi'an Jiaotong University Xi'an 710049 China

**Keywords:** amorphous‐crystalline phase transition, high‐entropy alloys, nucleation

## Abstract

High‐entropy alloys utilize high configurational entropy to stabilize solid solutions, suppress intermetallics, and broaden compositional possibilities. However, achieving homogeneity is challenging due to diffusion disparities, especially with refractory metals. Here, a unique kinetic pathway enabling the synthesis of a single‐phase FeCoNiCuNb alloy with 8.39 at.% Nb, is reported. In situ transmission electron microscopy heating experiments demonstrate that Cu and Nb exhibit significantly lower propensity to participate in solid solution formation compared to Fe, Co, and Ni. However, suppressing FeCoNi nuclei growth allows Cu/Nb incorporation into an amorphous phase with Fe, Co, and Ni. The nano‐sized FeCoNi nucleus undergoes dynamic transitions between crystalline and amorphous states, which increases the vacancy concentration and facilitates Cu and Nb diffusion, ultimately leading to the formation of FeCoNiCuNb nuclei. These phase transitions also enhance crystal growth during Ostwald ripening, ultimately yielding a stable single‐phase polycrystalline FeCoNiCuNb alloy. This study highlights unconventional kinetic strategies that expand phase diagram boundaries by leveraging the high‐entropy concept.

## Graphical abstract

The multi‐component crystal nucleus transitions between crystalline and amorphous states, generating vacancies that facilitate the incorporation of refractory elements. This process enables the formation of refractory high‐entropy alloys.

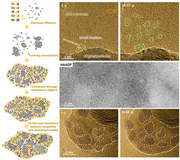

## 1 Introduction

Alloying has long been used to impart desirable properties to materials. To achieve improved performance, the secondary elements are often selected to be significantly different from the matrix elements. For example, refractory heavy elements such as tungsten (W), niobium (Nb), and tantalum (Ta) are commonly added to enhance the high‐temperature properties of alloys.^[^ ^1^, ^2^, ^3^ ^]^ However, the significant differences in atomic size and bonding characteristics between the matrix and secondary elements can result in low solubility, which often leads to the formation of complex intermetallic compounds.^[^ ^4^, ^5^, ^6^ ^]^ The more elements present in an alloy, the higher the likelihood that some will react to form these compounds. As a result, traditional alloying strategies are typically limited to narrow regions of phase diagrams—usually concentrated at the corners or edges. This restricts the number of possible element combinations and limits the alloying potential of conventional approaches.^[^ ^7^ ^]^

However, Yeh's hypothesis challenged this traditional view, proposing that when five or more elements coexist in near‐equiatomic proportions, the contribution of configurational mixing entropy to the free energy at the melting temperature becomes comparable to the formation enthalpies of strong intermetallic compounds.^[^ ^8^ ^]^ This suppresses the formation of most intermetallics, except those with particularly large negative heats of formation, such as ceramic compounds (oxides, carbides, nitrides, and silicides). As a result, random solid solutions are more easily formed during solidification. This concept led to the development of high‐entropy alloys (HEAs), which have gained significant attention in recent years. High‐entropy alloys offer new possibilities by breaking traditional alloy design rules and mixing multiple principal elements in relatively high, often equiatomic, concentrations. This new alloying strategy shifts the composition space to the center of the phase diagram for quinary and higher‐order multi‐component alloys, offering a far broader range of compositions and a significantly larger number of possible combinations than the narrow regions at the corners and edges of conventional phase diagrams.^[^ ^7^, ^9^, ^10^, ^11^, ^12^, ^13^, ^14^ ^]^

It is important to note that Yeh and co‐workers' conclusions are based on the assumption that the solid solution is ideal (random solution) and the competing intermetallic compound is perfectly ordered. However, it is widely accepted that while thermodynamics governs the final phase composition, kinetic processes control the distribution of phases and the resulting microstructure. The high‐entropy effect promotes the formation of solid‐solution phases and should be considered in thermodynamic models to determine the equilibrium structure and microstructure. However, the sluggish diffusion effect reduces the degree of homogeneity and slows phase transformation rates, which can influence phase formation as well.^[^ ^15^, ^16^ ^]^ Therefore, in practice, very few HEAs containing refractory heavy elements remain stable as solid solutions at all temperatures up to their melting points. Most HEAs investigated thus far tend to decompose into multiple solid phases under appropriate heat treatments.^[^ ^15^, ^17^, ^18^ ^]^ It is of great interest to explore whether it is indeed possible to form single‐phase high‐entropy alloys with high concentrations of refractory heavy elements and to identify the specific kinetic pathways that could facilitate their stability.

In this work, we successfully achieved the in situ growth of a FeCoNiCuNb solid solution containing 8.39 at.% Nb. Through in situ transmission electron microscope (TEM) heating experiments, we observed that the Fe, Co, and Ni atoms diffused more rapidly and tended to form a stable alloy phase, while Cu and Nb were less likely to participate in the solid solution phase formation. However, if the crystalline growth of the FeCoNi phase could be effectively suppressed, allowing the formation process to remain in the nucleation stage, Cu and Nb could be incorporated with Fe, Co, and Ni in an amorphous form. Importantly, the FeCoNi crystalline nuclei underwent continuous transitions between crystalline and amorphous structures, increasing the vacancy concentration, which facilitated the diffusion of Cu and Nb into the nuclei and enabled the formation of the FeCoNiCuNb nuclei. Such phase transformations between crystalline and amorphous states also facilitate further crystal growth during Ostwald ripening.

## 2 Results and Discussion

**Figure 1:**
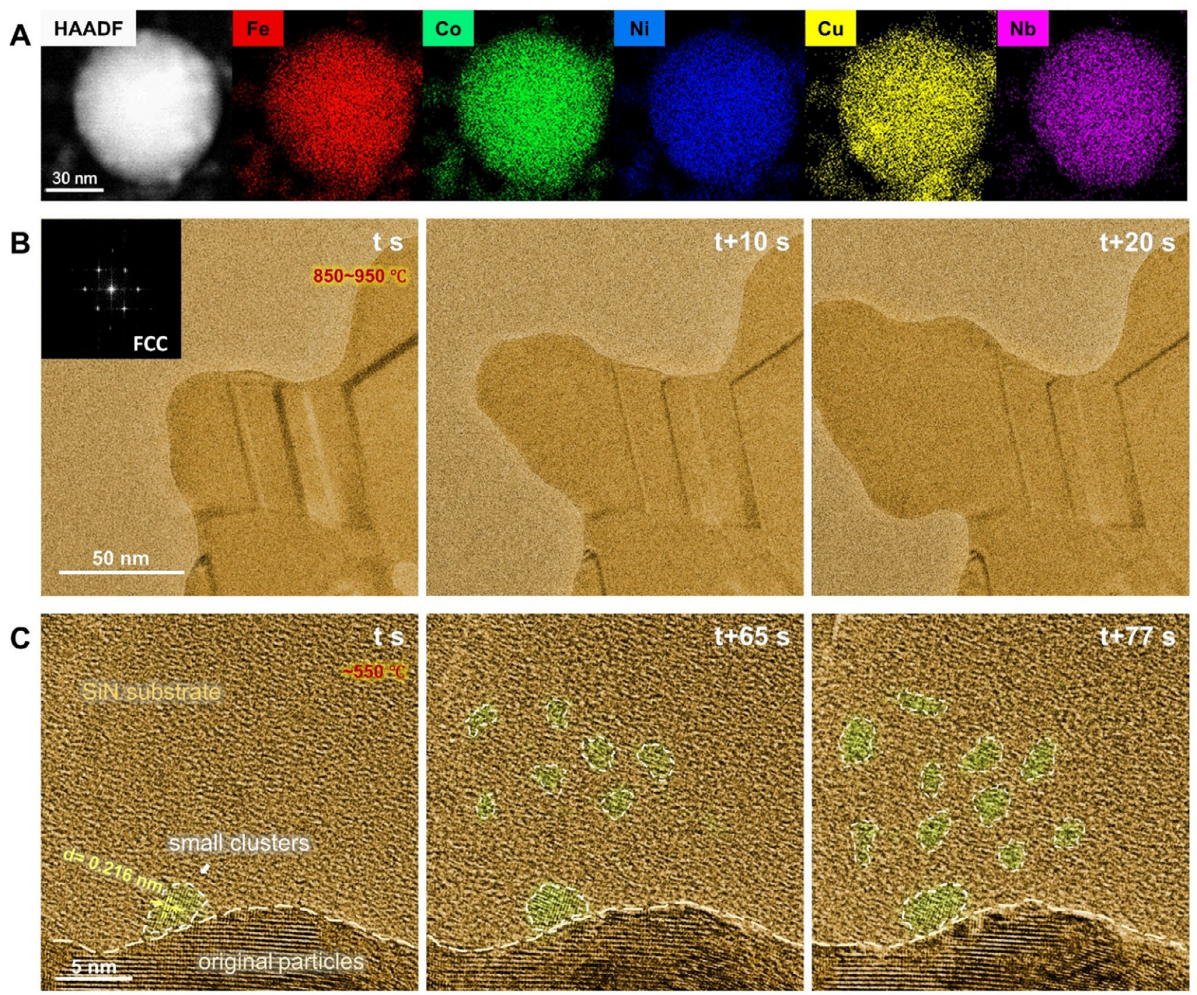
Elemental characterization of FeCoNiCuNb nanoparticles and in situ analysis of diffusion pathways. A) HAADF image of original NPs and corresponding EDS maps for individual elements of Fe, Co, Ni, Cu, and Nb. B) TEM frames of FeCoNi alloy growth from in situ Movie S1 (Supporting Information). C) TEM images taken at different times from in situ Movie S2 (Supporting Information) showing the process of diffusion‐driven formation of small atomistic clusters near the original particles. The original particle surface is outlined by the beige dashed line, while the small clusters are outlined by the white dashed line.

The in situ TEM experiments started with FeCoNiCuNb nanoparticles (NPs) synthesized by the arc discharge plasma method (details provided in Experimental Section). This preparation method enables the formation of a metastable phase, as it does not require strict thermodynamic equilibrium. We would like to know whether the FeCoNiCuNb solid solution alloy can continue to grow upon heating or if it will become thermodynamically unstable, causing certain elements to diffuse and form new alloys. The FeCoNiCuNb NPs used in the experiment contain 22 at.% Fe, 21 at.% Co, 21 at.% Ni, 25 at.% Cu, and 10 at.% Nb. Using spherical aberration‐corrected TEM (Spectra 300–30, operated at 300 kV), it can be observed that the original NPs (see Experimental Section) were predominantly spherical with diameters ranging from ≈50 to 150 nm, and the elemental distribution was relatively uniform, as shown in **Figure** **1**A.

In situ TEM heating experiments were conducted on the original NPs, employing various heating procedures to capture different microstructural evolution pathways. However, in most cases, the original FeCoNiCuNb NPs did not continue to grow into the FeCoNiCuNb solid solution. Instead, Fe, Co, and Ni atoms rapidly diffused out from the original metastable FeCoNiCuNb solution phase and immediately formed the FeCoNi solid solution phase. As shown in Figure 1B, the growth of the FeCoNi alloy occurred in all directions, resulting in complex microstructures, including grain boundaries and twin boundaries. The details of such microstructural evolution are provided in Movie S1 (Supporting Information), and the EDS of the newly formed region is shown in Figure S1 (Supporting Information), both as Supporting Information. This behavior aligns with prior research, which highlights that Cu has a positive mixing enthalpy with most elements.^[^ ^19^, ^20^ ^]^ Additionally, Nb, being a refractory element, has significant differences in size, electronegativity, and diffusion rate. During phase separation, Nb and Cu could not directly integrate into the FeCoNi lattice or participate in the crystal formation and growth. This indicates that, although the formation of FeCoNiCuNb crystals may be thermodynamically possible, it is not a straightforward kinetic process.

Nevertheless, we eventually identified one procedure that enabled the formation of the FeCoNiCuNb solid solution. In this procedure, the temperature was first rapidly raised to ≈300 °C, then gradually increased in 50 °C steps, maintaining each temperature for a period, while monitoring the real‐time changes in the original particles and the surrounding area. It was found that at ≈550 °C, atoms began diffusing out from the original particles, with some aggregating to form small atomistic clusters on the surface of the particles. The number of these clusters gradually increased over time. Figure 1C shows three representative moments of the diffusion process of small nanoscale clusters from the original particle (details in Movie S2, Supporting Information). At time *t*, a small atomic cluster, ≈3–5 nm in size, started to form at the edge of the original particle, with clear lattices and distinct orientation. The lattice spacing is ≈0.216 nm, close to the [111] spacing of FCC, but because the image only shows the orientation in one direction, the exact crystallographic orientation cannot be confirmed. As the temperature was maintained, the cluster continued to grow. By t + 65 s, another nanograin with a different orientation grew on the left side of this cluster. Meanwhile, an increasing number of nanosized clusters formed on the SiN substrate. Over time, the number of clusters increased, and their contrast in TEM mode became more pronounced. Overall, it was noted that at this stage, although the larger clusters displayed lattice fringes, the majority of the clusters were disordered in atomic structure. These clusters were typically irregular and non‐circular in shape, as indicated by the white dotted line in Figure 1C, suggesting that the early‐stage crystalline nuclei had a non‐equilibrium geometry.^[^ ^21^ ^]^ This is consistent with previous experimental studies that have identified fully or partially disordered states in the early stage of atomic crystallization in solution,^[^ ^22^, ^23^ ^]^ on surfaces,^[^ ^24^, ^25^ ^]^ or in nanomaterials.^[^ ^26^, ^27^ ^]^

**Figure 2:**
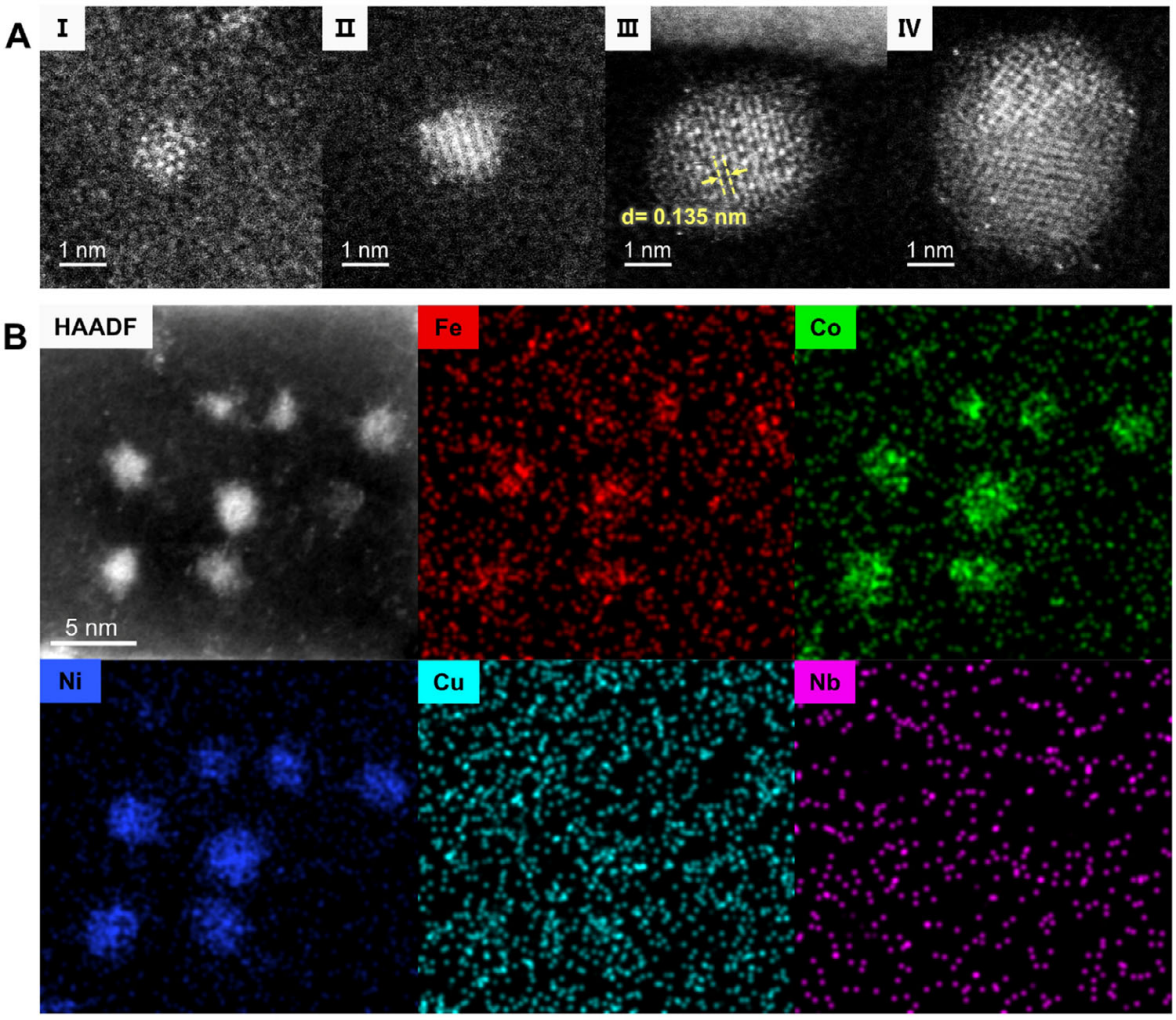
Structural and elemental characterization of newly formed nanoclusters. A) I–IV present HAADF‐STEM images of newly formed nanoclusters with different sizes. B) HAADF image of newly formed nanoclusters and corresponding EDS maps for individual elements of Fe, Co, Ni, Cu, and Nb.

To better characterize the atomic structure of the newly formed phase, we performed HAADF‐STEM characterization on the newly formed nanoclusters of different sizes. As shown in the HAADF‐STEM images in **Figure** **2**A, initially, these clusters may contain only a few atoms, in an irregular and disordered state (Figure 2A‐I). This phenomenon is common in clusters smaller than 1 nm. As they grew, more atoms were incorporated, and lattice structures gradually formed (Figure 2A‐II, III). For instance, the cluster shown in Figure 2A‐III began to display an FCC lattice. Some of the small clusters may also merge with nearby clusters, forming polycrystals (Figure 2A‐IV). Nevertheless, the HAADF‐STEM images reveal that the diffused clusters exhibited no specific growth direction, leading to varied crystal orientations and significant orientation differences between adjacent nuclei.

We were then curious about which elements were involved in the formation of the new nuclei and what the resulting alloy was. Therefore, we paused the in situ heating experiment by rapidly quenching the sample to room temperature and performed atomic‐resolution EDS analysis. The EDS analysis of atomic clusters in the early diffusion stage showed that Fe, Co, and Ni were notably enriched in the clusters (Figure 2B). Specifically, the very tiny clusters were enriched with Ni and Co only, and the Fe signal was also low. This indicates that Ni and Co diffused the fastest, and Fe atoms arrived later. Notably, it is easy to form NiCo alloys and FeCoNi alloys under these conditions. In contrast, although Cu and the refractory element Nb had also diffused out, their concentrations were much lower, and they were not involved in the formation of the nuclei. A small amount of these elements was observed as bright atomic spots around the clusters in Figure 2A‐III,IV, because they have higher atomic numbers than the other elements in the system.

**Figure 3:**
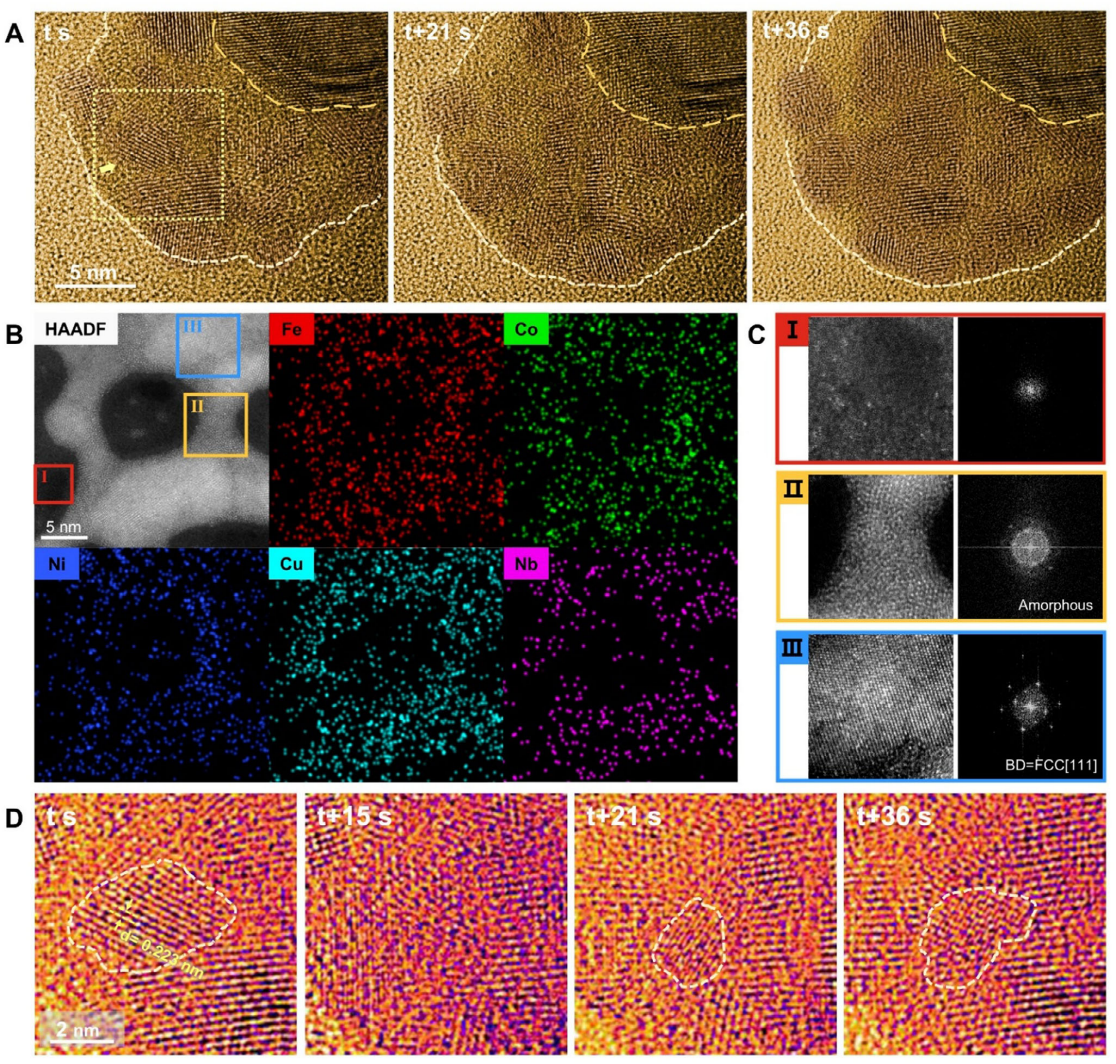
Formation and evolution of amorphous–crystalline hybrid structures during nucleation and early‐stage nanocluster assembly. A) TEM images taken at different times in in situ Movie S3 (Supporting Information) showing the formation of amorphous regions at the connections between individual crystal nuclei with different orientations. The original particle surface is outlined by the yellow dashed line, while the newly formed crystal nuclei and amorphous mixed regions are outlined by the beige dashed line. B) One typical HAADF‐STEM image of mixed structure and corresponding EDS maps for individual elements of Fe, Co, Ni, Cu, and Nb. C) The left side presents the magnified HAADF‐STEM images of the SiN substrate I), the amorphous structure at the connection II), and cluster regions III), corresponding to the red, yellow, and blue squares in B). The right side displays the corresponding FFT patterns. D) TEM images of one representative cluster (outlined by beige dashed lines), corresponding to the yellow dashed box area in A).

When the temperature was at 550 °C for 30 min, a large number of nano‐sized nuclei were formed. After the formation of these nuclei, the temperature was further increased in 10 °C increments, with each step held for 5 min. At ≈600 °C, it was observed that the amorphous thin film appeared and grew between small clusters, connecting the separated FeCoNi nuclei together and eventually forming an “amorphous egg‐white”– “Crystalline egg‐yolk” mixed structure. **Figure** **3**A shows a series of HRTEM images captured during an in situ HRTEM heating experiment, clearly demonstrating the formation of amorphous regions at the connections between individual crystalline nuclei with different orientations (details in Movie S3, Supporting Information). From t to t + 36 s, the amorphous area expanded, and simultaneously, the size of the crystalline clusters changed.

This amorphous film with embedded nano‐sized crystalline clusters can grow to over several hundred nanometers in all directions. Figure 3B shows a typical HAADF‐STEM image of this structure and its corresponding EDS mapping. The left side of Figure 3C presents the magnified HAADF‐STEM images of the SiN substrate I), the amorphous structure at the connection II), and cluster regions III), corresponding to the red, yellow, and blue squares in Figure 3B. It was noted that individual bright dots were observed on the nearby substrate, for example, in area I, indicating the existence of single atoms around. The right images in Figure 3C show the corresponding FFT patterns, clearly demonstrating the structures of the amorphous and crystalline clusters, respectively. Interestingly, the EDS maps of the mixed amorphous and crystalline region indicated that all five elements—Fe, Co, Ni, Cu, and Nb—were present and appeared to be uniformly distributed. This contrasts sharply with the EDS analysis of the earlier‐formed crystalline nuclei in Figure 2B in which Cu and Nb were not detected. This suggests that Cu and the refractory element Nb were successfully integrated during the growth of the FeCoNiCuNb amorphous structure. The concentrations of Fe, Co, Ni, Cu, and Nb, at this stage, were measured to be 17, 22, 30, 25, and 6 at.%.

Through careful analysis of the in situ structural evolution at the atomic scale, we found that the lattice orientation of these clusters kept changing during this process. To explore the details of these changes, we analyzed the structural evolution of one representative cluster. Figure 3D shows a series of magnified TEM images of the region where the cluster (outlined by beige dashed lines) is located, corresponding to the yellow dashed box area in Figure 3A. At time t, the clusters exhibited clear lattice fringes with a spacing of 0.223 nm, characteristic of a $\left[11\bar{1}\right]$ in the FCC structure. However, after 15 s, the lattice disappeared, and the iFFT image also revealed a disordered state. By 21 s, crystalline lattice fringes reappeared with a different orientation ($\left[13\bar{1}\right]$ FCC) and a different cluster size, at a location that is not exactly the same as where it disappeared. Over the next few seconds, the state continued to fluctuate, alternating between crystalline and disordered states until 36 s, when more distinct lattice fringes reemerged.

Such changes in lattice orientation are distinct from the phenomena of grain rotation observed in previous literature.^[^ ^28^, ^29^ ^]^ Here, the transitions were abrupt rather than continuous, and both the size and location of the crystalline nuclei changed. It is noted that generally, the free energy of the transition from crystal to amorphous is high, but the free energy required for the transition from disordered to crystalline state is negative and large enough to drive the transition spontaneously. However, when the clusters are small (≈2.0 nm in their study), the energy and barrier between the two states are minimal, allowing the low energy barrier for transitioning from crystalline to disordered states to be easily overcome by various sources.^[^ ^30^ ^]^ Specifically in high‐entropy systems, the high‐entropy effect may further reduce the energy difference and potential barrier between crystallization and disorder.^[^ ^31^, ^32^, ^33^ ^]^ The diffusion of a species occurs faster in amorphous structures, which are more open than crystal structures of the same composition. Such transitions at high temperatures may result in the increase of vacancy concentration,^[^ ^34^ ^]^ which facilitates the diffusion of Nb and Cu in the crystalline nucleus. This is a critical event that enables the formation of FeCoNiCuNb nuclei.

**Figure 4:**
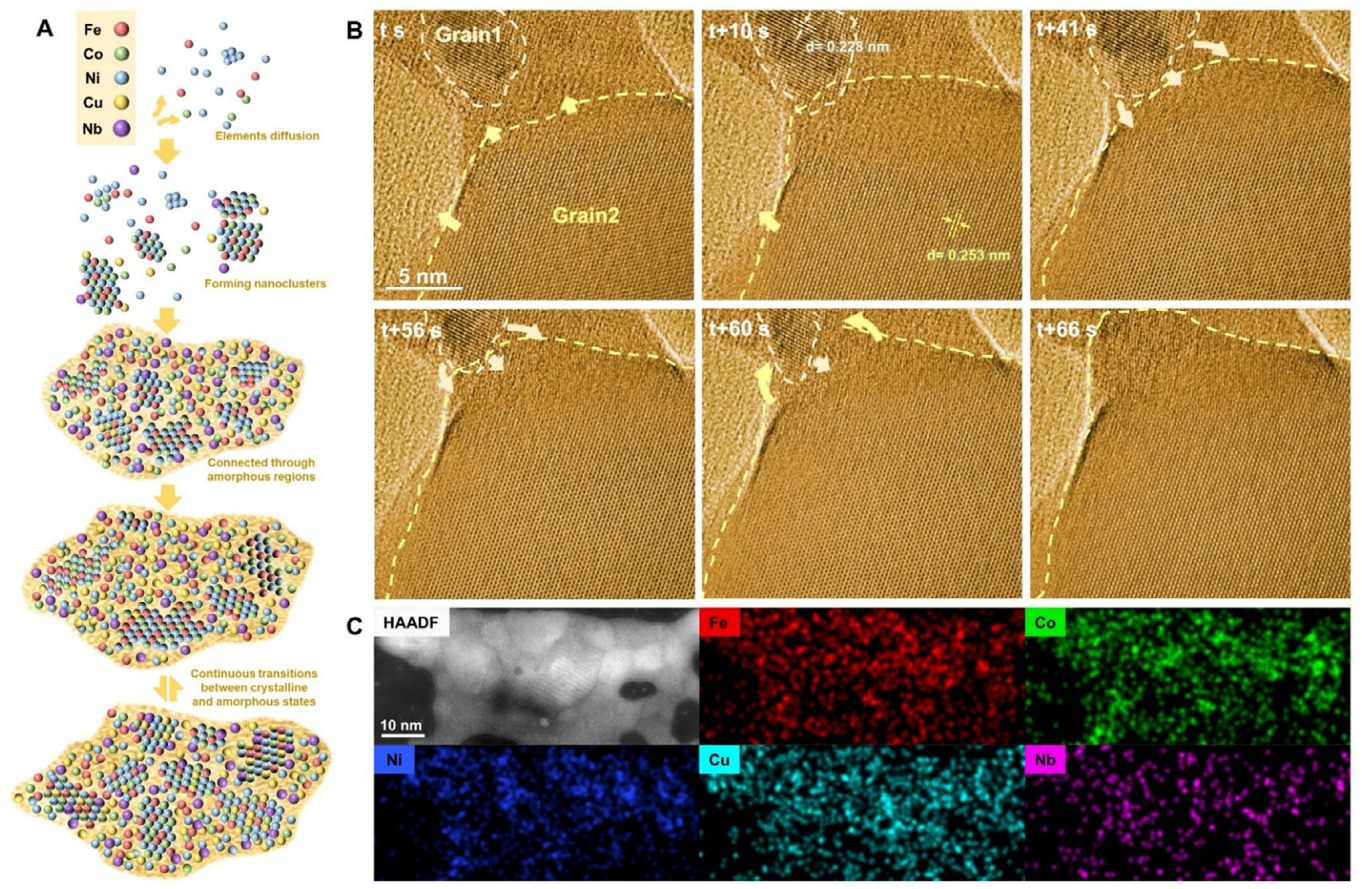
Mechanism and evolution of crystal nucleation and growth in achieving solid solution alloy formation. A) The schematic diagram of the crystal nucleation process. B) TEM images taken at different times in in situ Movie S4 (Supporting Information) showing a typical process of the crystal growth. The beige‐dotted circle denotes Grain 2 (G2), with the beige arrow indicating its growth direction. Similarly, the yellow‐dotted circle represents Grain 1 (G1), with the yellow arrow showing its growth direction. C) HAADF‐STEM image of a newly formed polycrystalline FeCoNiCuNb solid solution alloy and corresponding EDS maps for individual elements of Fe, Co, Ni, Cu, and Nb.

**Figure** **4**A is a schematic diagram illustrating the crystal nucleation process described above. As the temperature rises, Fe, Co, and Ni diffuse out first, forming early‐stage irregular clusters. Cu, Nb, and the remaining Fe, Co, and Ni subsequently form an amorphous phase that gradually grows and connects the FeCoNi nuclei, creating a mixed structure of crystalline nuclei and amorphous regions. Under certain processing controls, the growth of these nuclei can be suppressed. The nuclei remain unstable and undergo continuous transformations between crystalline and amorphous states. Such transformations increase the vacancy concentration, facilitating the diffusion of Cu and Nb into the nuclei.

The nuclei exhibit varied shapes, sizes, and orientations. Upon further heating, the nuclei continued to grow. However, the growth rates of individual nuclei vary. Once the nuclei reach a critical size, the structures and lattice orientations become stable, and the growth of the FeCoNiCuNb alloy can be observed. The growth process clearly followed a typical pattern of “big one eats small ones.” Interestingly, the detailed observations of the “digestion” process also revealed transitions between the crystalline and amorphous phases. Figure 4B illustrates a representative process of the subsequent crystal growth (details in Movie S4, Supporting Information). The crystalline orientation of Grain1 (G1) was indexed as $\left[1\bar{1}1\right]$, and Grain2 (G2) was indexed as [220]. At time *t*, G1 and G2 were connected by an amorphous region, and both were growing continuously. At t + 10 s, G2 grew upward and got in touch with G1. The two grains then came into contact. At t + 56 s, part of the G1 became amorphous with only 1/3 of the original G1 remaining crystalline. Until t + 66s, G1 had completely become an amorphous state and became the frontier of G2. This disordered region subsequently crystallized into the same structure and orientation as G2.

Ultimately, a polycrystalline FeCoNiCuNb solid solution alloy can be formed in this way. The EDS mapping demonstrates that all elements are uniformly distributed within the newly formed alloy, with the Nb concentration reaching 8.39 at.%, as shown in Figure 4C. The results demonstrate that it is indeed possible to incorporate high concentrations of refractory elements into high‐entropy alloys without producing secondary phases, a phenomenon rarely observed in traditional alloy systems.

Furthermore, we designed a series of heating schemes and experimentally confirmed that a single‐phase FeCoNiCuNb solid solution can be obtained as long as a key kinetic condition is met: within the temperature window of 550–600 °C, the heating rate must be sufficiently slow to suppress the growth of the competing FeCoNi phase. This was confirmed by additional experiments using alternative procedures (e.g., direct heating to 550 °C with isothermal holding; stepwise heating with 50 °C increments up to 550 °C followed by holding; and stepwise heating with 10 °C increments up to 550 °C followed by holding). In all cases, nanoclusters diffused during the isothermal hold near 550 °C and underwent similar subsequent transformations, ultimately forming a single‐phase solid solution.

We also conducted additional in situ heating experiments using carbon films at lower heating temperatures. The observed diffusion behavior was essentially identical to that on SiN membranes, as shown in Figure S2 (Supporting Information). Furthermore, we performed additional in situ heating experiments at 200 kV (details in Figure S3A, Supporting Information), which revealed the same structural evolution observed at 300 kV. Furthermore, in situ‐heating experiments were performed with the electron beam off, followed by post‐heating imaging at 300 kV (details in Figure S3B, Supporting Information). The results were consistent with those from direct 300 kV observations.

## 3 Conclusion

Our study demonstrates that although the growth of an FeCoNiCuNb single‐phase FCC alloy containing a large amount of the refractory element Nb can be achieved, the kinetic pathway is complex, as the direct nucleation of the FeCoNiCuNb nuclei proved difficult. Instead, the FeCoNi nuclei form first and gradually “digest” Cu and Nb through the phase transformations between crystalline and amorphous states, resulting in the formation of FeCoNiCuNb nuclei. The successful nucleation of the FeCoNiCuNb quinary single‐phase alloy is closely related to the following two key factors: 1) a well‐designed heating process that suppresses the growth of FeCoNi nuclei, and 2) the continuous transitions of the nuclei between amorphous and crystalline states, which produce vacancies that facilitate diffusion of Cu and Nb into the nuclei, enabling the formation of FeCoNiCuNb nuclei. This process is strongly linked to the high‐entropy system since a more complex composition generally increases the critical size for crystalline nucleation due to thermodynamic and kinetic factors. The critical size for nucleation (γ*) is given by the classical nucleation theory:

(1) $$\gamma^{*}=\frac{2\gamma }{\Delta G_{v}}\;$$

where γ*is the interfacial energy between the nucleus and the surrounding matrix, and ΔG_v_ is the Gibbs free energy change per unit volume. A more complex composition often leads to a greater variety of atomic species and bonding environments, creating local variations in energy and atomic arrangement. This makes it harder for a stable crystalline nucleus to form. In addition, the thermodynamic driving force for nucleation (ΔG, the Gibbs free energy change) may be reduced due to competing interactions between different elements.

**Figure 5:**
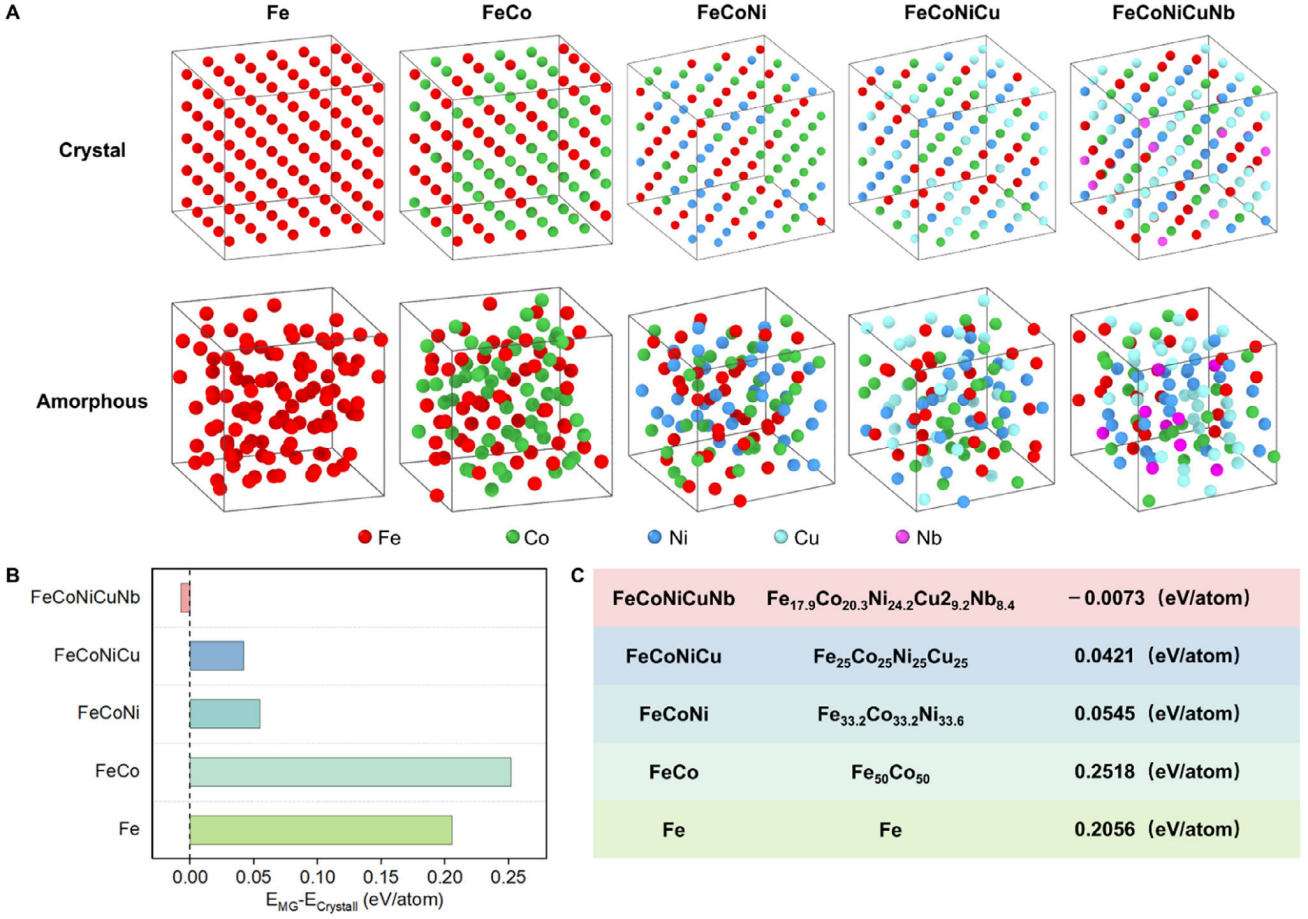
Relative thermodynamic stability of crystalline and amorphous phases in multiple alloy systems revealed by density functional theory (DFT) calculations. A) Atomic configurations of the crystalline (top row) and metallic glass (MG, bottom row) phases for Fe, FeCo, FeCoNi, FeCoNiCu, and FeCoNiCuNb alloys. The stable crystalline phases were constructed in BCC (Fe, FeCo) or FCC (others) lattices, while amorphous configurations were obtained via ab initio molecular dynamics (AIMD) simulations by rapid melt quenching. Atoms are color‐coded as follows: Fe (red), Co (green), Ni (blue), Cu (cyan), and Nb (purple). B) DFT‐calculated energy difference (E_MG_−E_Crystall_) between the MG and crystalline state for the five alloy systems. C) Specific atomic compositions of the five alloy systems, together with the energy difference (E_MG_−E_Crystall_).

To substantiate this, we performed additional density functional theory (DFT) calculations to compare the energy differences between crystalline and amorphous states for a series of alloy systems with increasing compositional complexity (pure Fe, FeCo, FeCoNi, FeCoNiCu, and FeCoNiCuNb). The amorphous configurations were generated by rapid quenching of the corresponding melts using ab initio molecular dynamics (AIMD) simulations (details provided in Experimental Section). **Figure** **5**A shows atomic configurations of the crystalline and amorphous phases for Fe, FeCo, FeCoNi, FeCoNiCu, and FeCoNiCuNb alloys. As shown in Figure 5B, FeCoNiCuNb exhibits an amorphous structure that is energetically more stable than its crystalline counterpart, whereas the other systems (Fe, FeCo, FeCoNi, and FeCoNiCu) favor the stable crystalline phase (BCC for Fe and FeCo; FCC for FeCoNi and FeCoNiCu). Notably, the relative energy difference decreases markedly with increasing compositional complexity, reaching ≈ −0.01 eV atom^−1^ in FeCoNiCuNb—much lower than that in FeCo (0.25 eV atom^−1^), as shown in Figure 5C. In fact, a dramatic decrease of the energy barrier is observed when the alloying system changes from FeCo to FeCoNi. This reduction indicates that amorphous–crystalline fluctuations become kinetically accessible in multicomponent systems, thereby providing a thermodynamic driving force such as Nb incorporation. This behavior originates from the high‐entropy effect, which simultaneously enhances thermodynamic stability while destabilizing the crystalline phase, reducing the free energy difference between the amorphous and crystalline states. Coupled with the sluggish diffusion, a larger critical nucleus size is required to overcome the energy barrier for nucleation, prolonging the nucleation stage. Consequently, the initial clusters remain at a small size for an extended period, allowing them to transition easily between crystalline and amorphous phases. This process generates vacancies that promote the incorporation of Nb and Cu into the nucleus.

Therefore, thermodynamically, our experimental and computational results demonstrate that the compositional complexity of an alloy exerts a pronounced influence on the free energy difference between the crystalline and amorphous states. This allows us to propose an extended compositional design strategy: for alloy systems that require the incorporation of immiscible elements while still targeting a single‐phase solid solution, the reversible crystalline–amorphous transition and compositional evolution observed during nucleation offer a new opportunity to achieve both objectives. We also acknowledge that configurational entropy is a key but not the only influencing factor; the behavior of real alloys is far more complex. The development of a fully generalized thermodynamic design strategy will require further systematic efforts.

Nevertheless, our study demonstrates the feasibility of forming a single‐phase alloy with high concentrations of refractory elements by leveraging the high‐entropy concept. Additionally, it unveils the intricate and unique kinetic pathway required to achieve this outcome. Specifically, the multi‐component crystal nucleus can transition between crystalline and amorphous states, generating vacancies that facilitate the incorporation of refractory elements. This process enables greater incorporation of refractory elements into the alloy systems.

## 4 Experimental Section

### Alloy Preparation

The FeCoNiCuNb NPs were synthesized using an arc‐discharge plasma method. Initially, high‐purity metal powders of each constituent element were mechanically mixed and compressed into cylindrical targets, which were then placed in the anode region of a direct current (DC) arc‐discharge chamber. The chamber was evacuated to a vacuum level of 10^−3^ Pa, then filled with hydrogen (H_2_) as the reaction gas and argon (Ar) as the protective gas, achieving a stabilized pressure of 3 × 10^4^ Pa. The arc‐discharge process was maintained for 5–10 min. After the discharge, the synthesized NPs underwent a 6‐h passivation process within the chamber before being collected.

### TEM Characterizations and In Situ Heating Experiments

The FeCoNiCuNb NPs for characterization were ultrasonically dispersed in ethyl alcohol for 10 min and then deposited onto a molybdenum grid to prepare TEM samples. The microstructure was analyzed using TEM, (Tecnai G2 F20 S‐TWIN, FEI) and spherical‐aberration‐corrected TEM (Titan G2 60–300, FEI, operated at 200 kV, and Spectra 300–30 operated at 300 kV). Energy‐dispersive X‐ray spectroscopy (EDS) analysis was conducted using the Spectra 300–30 microscope equipped with a Super‐X EDS detector.

For in situ TEM heating experiments, the FeCoNiCuNb NPs were dispersed onto SiN membrane substrate in microelectromechanical‐systems‐based heating chips (Nanochip, DENS solutions) following previously reported methods. The experiments were performed using a double‐tilt heating holder (Wildfire D6, DENS solutions). Both conventional TEM instruments (Tecnai G2 F20 S‐TWIN, FEI) and spherical‐aberration‐corrected TEM instruments (Titan G2 60–300, FEI, and Spectra 300–30) were used for these characterizations and in situ studies.

### Density Functional Theory (DFT) Calculation

The DFT calculation was performed to evaluate the energy differences between crystalline and amorphous states for a series of alloy systems of increasing compositional complexity: pure Fe, FeCo, FeCoNi, FeCoNiCu, as well as the quinary high‐entropy alloy Fe_17_._70_Co_20_._28_Ni_24_._40_Cu_29_._22_Nb_8_._39_ (denoted FeCoNiCuNb) and Fe_17_._70_Co_20_._28_Ni_24_._40_Cu_29_._22_Zn_8_._39_ (denoted FeCoNiCuZn) using the Vienna Ab Initio Simulation Package (VASP).^[^ ^35^ ^]^ The crystalline phases were constructed in body‐centered cubic (BCC) lattices for Fe and FeCo, and face‐centered cubic (FCC) lattices for FeCoNi, FeCoNiCu, and the two quinary alloys. A random substitution method was used to generate atomic configurations that approximate the random solid‐solution nature of high‐entropy alloys. Supercells contained either 256 atoms (FCC) or 250 atoms (BCC), and Brillouin zone sampling was performed using a 2 × 2 × 2 Monkhorst–Pack k‐point mesh centered at Γ. The lattice constants were optimized by fitting pressure–volume curves to ensure a near‐zero residual pressure. The amorphous structures were generated via ab initio molecular dynamics (AIMD) by rapidly quenching the molten alloys. Each system was first equilibrated at 2500 K for at least 10 ps in the isothermal (NVT) ensemble to obtain a homogeneous liquid. A stepwise cooling protocol was then applied, decreasing the temperature from 2500 to 300 K in 300 K intervals at a rate of 50 K ps^−1^. At each temperature step, the structure was equilibrated for 10 ps at 0 GPa. The amorphous supercells consisted of 108 atoms, and a 3 × 3 × 3 Monkhorst–Pack k‐point mesh was employed for Brillouin zone integration. The generalized gradient approximation (GGA) with the Perdew–Burke–Ernzerhof functional was employed to describe the exchange‐correlation interactions, and the projector‐augmented wave (PAW) method was used.^[^ ^36^, ^37^ ^]^ Valence‐electron wavefunctions were expanded in a plane‐wave basis set, with a kinetic energy cutoff of 380 eV. Final energies were obtained after conjugate gradient (CG) structural relaxation at 300 K. The reported energy differences between the metallic glass and crystalline states are based on these fully relaxed structures.

## Conflict of Interest

The authors declare no conflict of interest.

## Supporting information



Supporting Information

Supplemental Movie 1

Supplemental Movie 2

Supplemental Movie 3

Supplemental Movie 4

Supplemental Movie 5

## Data Availability

The data that support the findings of this study are available from the corresponding author upon reasonable request.
