# Supplementary material for: Phase Transformation‐Assisted Nucleation and Growth of a Single‐Phase FeCoNiCuNb Alloy
Source: Adv Sci (Weinh). 2025 Nov 6;13(5):e09237. doi: 10.1002/advs.202509237 (PMC12849958; doi:10.1002/advs.202509237)
Supplement: Supplementary file 1 — Supporting Information [file ADVS-13-e09237-s002.docx]

Supporting Information

**Phase Transformation-Assisted Nucleation and Growth of a Single-Phase**

**FeCoNiCuNb Alloy**

Zhimin Guo, Fuchen Zhou, Jinhua Yu, Jun Ding, Evan Ma, Qian Yu*

**Supporting Information includes:**

Fig. S1 to S3

Movie S1 to S5


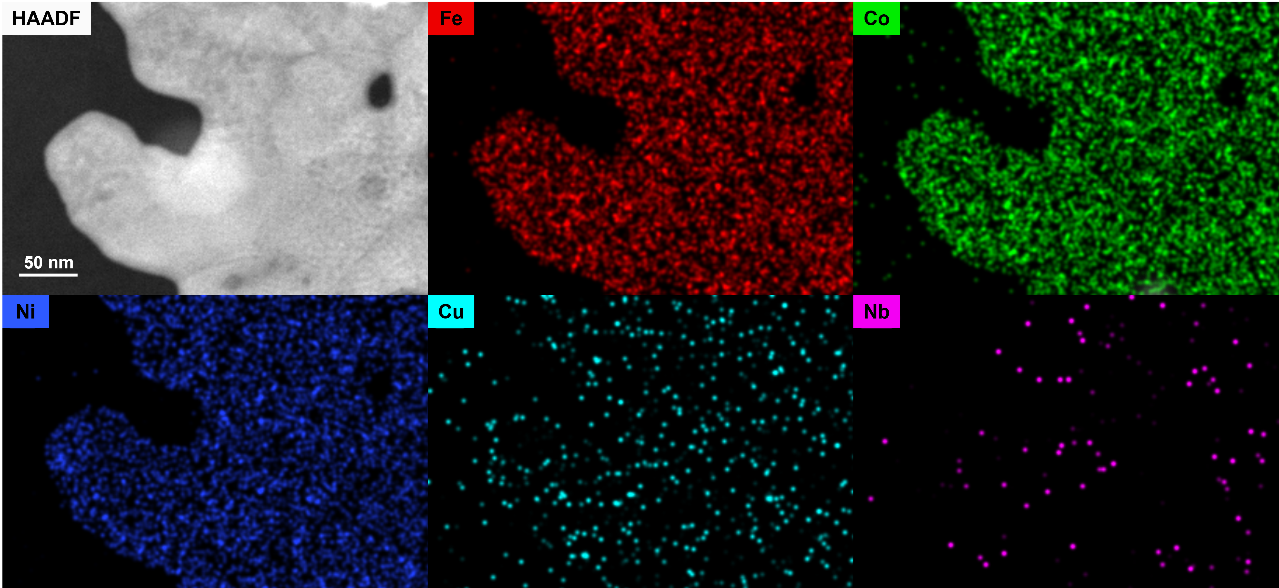


Figure S1.

**HAADF image of the newly formed region and corresponding EDS for individual elements of Fe, Co, Ni, Cu and Nb.**


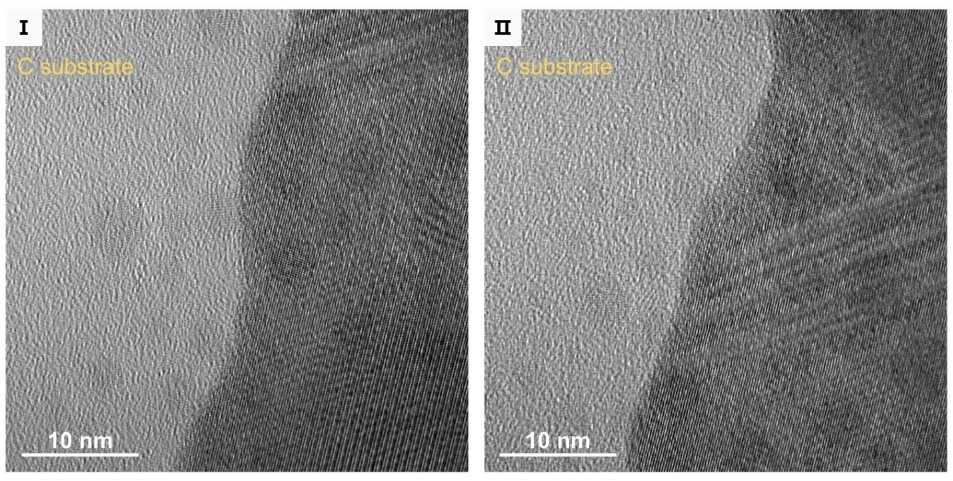


**Figure S2.**

**TEM images showing the diffusion of small clusters during in-situ heating experiments using a carbon (C) film as the substrate.**


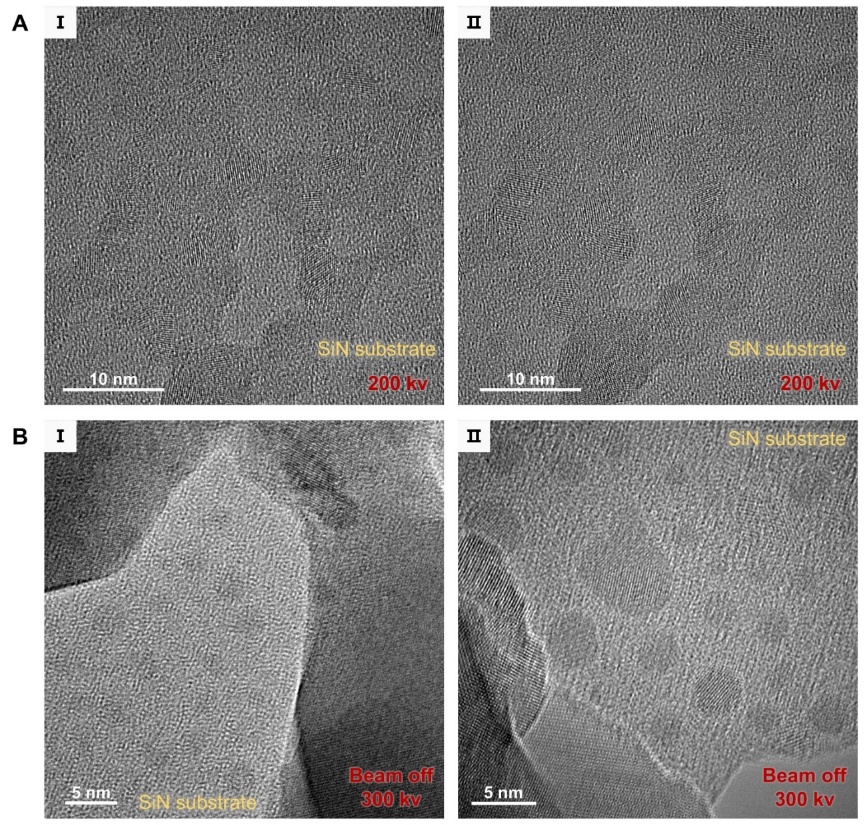


**Figure S3.**

**Assessment of electron beam effects during in situ heating of FeCoNiCuNb alloys.** **A**) TEM images at 200 kV acceleration voltage showing the formation of amorphous regions at the connections between individual crystal nuclei with different orientations during in-situ heating experiments. **B**) In situ heating experiments were performed in TEM with the electron beam turned off, and TEM images were subsequently taken at 300 kV acceleration voltage after the heating was completed.

**Movie S1.**

The growth of the FeCoNi alloy (speed up to two times).

**Movie S2.**

The diffusion process of small nanoscale clusters from the original FeCoNiCuNb particle (speed up to two times).

**Movie S3.**

The formation of amorphous regions at the connections between individual crystal nuclei with different orientations (speed up to two times).

**Movie S4.**

A typical process of the FeCoNiCuNb alloy growth (speed up to three times).

**Movie S5.**

The diffusion process of small nanoscale clusters from the original FeCoNiCuZn particle (speed up to three times).
